# Predictors of pre-resection hydrocephalus in posterior cranial fossa tumors: development of a predictive scoring model

**DOI:** 10.1007/s10143-025-03752-2

**Published:** 2025-08-19

**Authors:** Piriya Kanjanakangwankul, Bunpot Sitthinamsuwan, Chanon Ngamsombat, Chottiwat Tansirisithikul, Sarun Nunta-aree

**Affiliations:** 1 Division of Neurosurgery, Department of Surgery, Faculty of Medicine Siriraj Hospital, Mahidol University, 2 Wang Lang Road, Bangkok Noi, Bangkok, 10700 Thailand; 2 Department of Surgery, Phrachomklao Petchaburi Hospital, Petchaburi, Thailand; 3 Department of Radiology, Faculty of Medicine Siriraj Hospital, Mahidol University, Bangkok, Thailand

**Keywords:** Clival angle, Hydrocephalus, Posterior cranial fossa tumor, Posterior cranial fossa volume, Predictor, Pre-resection, Straight sinus angle, Tumor volume

## Abstract

Brain tumors located in the posterior cranial fossa frequently lead to hydrocephalus. However, few investigations have delineated factors predicting preoperative hydrocephalus. This study aimed to identify risk factors for hydrocephalus prior to tumor resection in the posterior cranial fossa and to develop a predictive scoring model. We reviewed 421 patients with posterior cranial fossa tumors, stratifying them into groups with (*n* = 160, 38%) and without (*n* = 261, 62%) hydrocephalus on initial neuroimaging. Demographic, radiographic, and craniometric variables were collected. Univariate and multivariable logistic regression analyses were performed, and odds ratios with 95% confidence intervals were determined. Factors that remained significant in the multivariable model were incorporated into a novel scoring system. Multiple factors were associated with preoperative hydrocephalus in univariate analysis. In the final multivariable model, increased intracranial pressure, ataxia, cognitive impairment, large tumor volume, and peritumoral vasogenic edema showed strong correlations with hydrocephalus. Pre-resection hydrocephalus in patients with posterior cranial fossa tumors was strongly associated with clinical symptoms (increased intracranial pressure, ataxia, and cognitive impairment) and radiographic findings (large tumor volume and peritumoral vasogenic edema). These results may guide early surveillance and facilitate surgical prioritization to avert severe complications in this patient population.

**Supplementary Information:**

The online version contains supplementary material available at 10.1007/s10143-025-03752-2.

## Introduction

Posterior cranial fossa lesions are frequently encountered in neurosurgical practice, with neoplasms constituting a major subset [1]. These tumors affect a broad range of age groups [2, 3]. In adults, meningiomas and schwannomas are common posterior fossa tumors [2, 3], whereas pilocytic astrocytomas, medulloblastomas, ependymomas, and diffuse midline gliomas predominate in pediatric patients [2–6]. Malignant gliomas and lymphomas typically arise in the supratentorial region rather than the posterior fossa [7]. Other tumors in this region include metastases, hemangioblastomas, and glioneuronal neoplasms [8–11]. Owing to the confined space of the posterior cranial fossa, these lesions often obstruct cerebrospinal fluid (CSF) flow, causing hydrocephalus [12].

In neurosurgical practice, hydrocephalus is a frequent complication of posterior fossa tumors, particularly in children [13–15]. It may result from impaired CSF pathways, overproduction of CSF, or a combination of both [16–20]. Tumors strongly associated with hydrocephalus include hemangioblastomas, large vestibular schwannomas, and pediatric brain tumors [21–24]. Surgical resection remains the primary treatment, as it is relatively safe, effective in reducing mass effect, and associated with favorable outcomes [25–28]. In patients with coexisting hydrocephalus, presurgical or intraoperative CSF diversion may be necessary [29–33]. Resection can often reestablish CSF flow, thus reducing the risk of persistent postoperative hydrocephalus and the need for permanent CSF diversion [34].

Although large posterior fossa tumors frequently cause hydrocephalus, some patients with small-to-medium tumors also develop this condition, suggesting additional contributing factors. Most studies have focused on risk factors for postoperative or persistent hydrocephalus, with few investigating predictors of hydrocephalus before tumor resection. This study therefore aimed to identify factors associated with pre-resection hydrocephalus in patients harboring posterior cranial fossa tumors and to develop a predictive scoring model.

## Materials and methods

### Study design and ethical approval

This cross-sectional study was ethically approved by Institutional Review Board (certificate of approval no. Si 975/2021). All patients with tumors arising in the posterior cranial fossa, treated at our medical institute from January 2013 to December 2020, were included into the study.

### Patient selection and exclusion criteria

Eligible patients had tumors confined to the posterior cranial fossa. Those with multifocal brain tumors, tumors involving both infratentorial and supratentorial regions, prior brain surgery, prior cranial radiation therapy or radiosurgery, or radiographic evidence of hydrocephalus before tumor diagnosis were excluded. Patients with a known history of hydrocephalus preceding brain tumor development were also excluded.

### Radiographic criteria for hydrocephalus

Patients were grouped according to the presence or absence of hydrocephalus on preoperative cranial magnetic resonance imaging (MRI). Hydrocephalus was diagnosed if one or more of the following were present [35–37]:

- Evans’ index > 0.3.
- Dilation of the third ventricle and lateral ventricles.
- Temporal horn dilation ≥ 5 mm on axial images.
- Thinning and elevation of the corpus callosum.
- Periventricular white matter hyperintensity on T2-weighted or fluid-attenuated inversion recovery (FLAIR) MRI.

### Data collection

Collected data were categorized into demographic, radiographic, and craniometric factors.

**Fig. 1:**
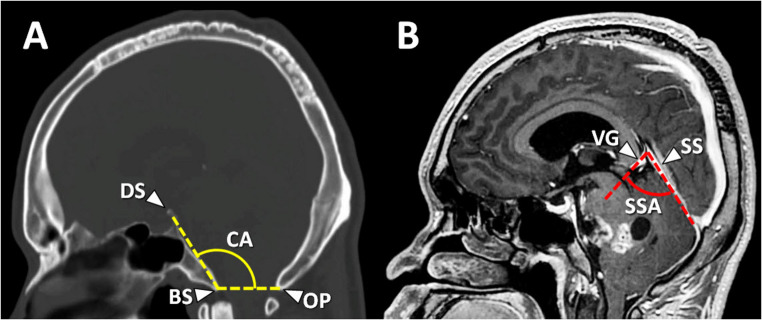
Craniometric analysis on mid-sagittal imaging. (**A**) The clival angle (CA) is measured as the angle formed between the top of the dorsum sellae (DS), basion (BS), and opisthion (OP). (**B**) The straight sinus angle (SSA) is defined by the angle created where the vein of Galen (VG) enters the straight sinus (SS)

- *Demographic factors*: The demographic factors were age, sex, duration of presenting symptoms, presenting symptoms, and tumor pathology. The presenting symptoms were categorized into increased intracranial pressure (IICP) symptom, ataxia, cognitive impairment, deterioration of consciousness, and cranial nerve dysfunction.
- *Radiographic factors*: Tumor volume, tumor location, involvement of posterior fossa neural structures, post-contrast tumor enhancement, peritumoral vasogenic edema, and intratumoral cystic appearance were evaluated. All radiographic data were retrieved from presurgical cranial MRIs in Synapse PACS (Fujifilm Healthcare).
- *Craniometric factors*: Craniometric measurements included the clival angle (Boogard’s angle), the straight sinus angle, and the posterior cranial fossa volume. The clival angle was defined by the angle connecting the dorsum sellae, basion, and opisthion on mid-sagittal imaging (Fig. 1A) [38]. The straight sinus angle was measured by the angle formed where the vein of Galen enters the straight sinus on mid-sagittal imaging (Fig. 1B) [39].

### Measurement procedures

**Fig. 2:**
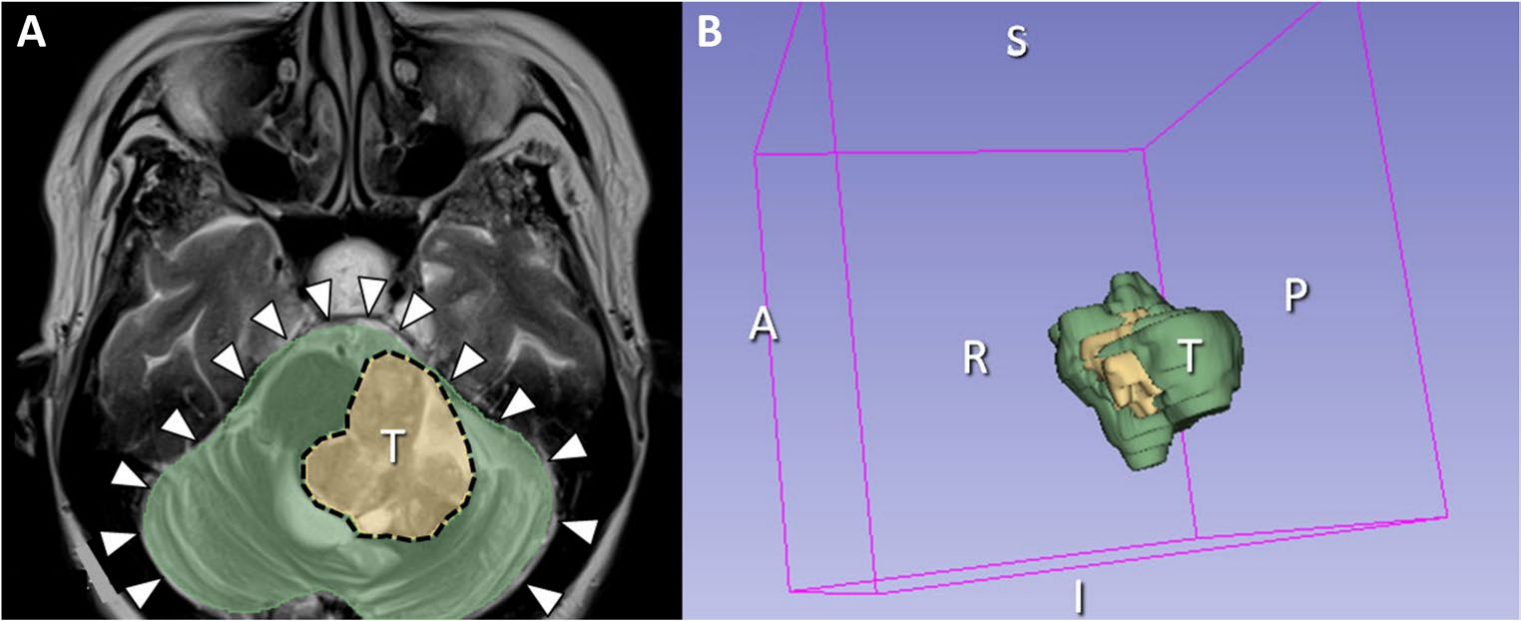
Tumor and posterior cranial fossa volume analysis using 3D Slicer software. (**A**) Axial T2-weighted magnetic resonance imaging illustrating the outlined measurements of tumor volume (dotted contour) and posterior cranial fossa volume (arrowheads). (**B**) Three-dimensional reconstruction of tumor shape and volumetric assessment using 3D Slicer software. Abbreviations: A, anterior; I, inferior; P, posterior, R, right; S, superior; T, tumor

Tumor and posterior cranial fossa volumes were quantified using 3D Slicer software [40, 41] (Fig. 2). Methods of 3D volumetric analysis followed published protocols [42, 43]. A neurosurgeon (P.K.) performed all measurements, which were independently confirmed by a neuroradiologist (C.N.).

### Statistical analysis

All data analyses were performed using PASW Statistics, version 18.0 (SPSS Inc, Chicago, IL, USA). Categorical variables (sex, clinical symptoms, and tumor characteristics) are presented as frequencies and percentages. Continuous variables (age, duration of presenting symptoms, tumor volume, posterior cranial fossa volume, clival angle, and straight sinus angle) are reported as mean with standard deviation or median with range, depending on the normality test.

### Univariate analysis

The association between each individual variable and the presence of pre-resection hydrocephalus was evaluated using Pearson’s chi-square or Fisher’s exact test for categorical data. The independent samples *t*-test or Mann‒Whitney *U* test was used to compare continuous variables between the hydrocephalus and non-hydrocephalus groups. The strength of association was quantified by odds ratio (OR) with a 95% confidence interval (95% CI). Statistical significance was set at *p* < 0.05.

### Multivariable analysis and assessment of collinearity

Variables with *p* < 0.2 in univariate analysis were selected for further evaluation. Multiple linear regression was conducted to identify collinearity, defined as tolerance < 0.2 and variance inflation factor > 5. Parameters that met these thresholds were excluded from subsequent binary logistic regression.

### Model development and validation

Backward (Wald) selection was applied during binary logistic regression to derive the final predictive model, using a probability for removal of 0.05. Bootstrap internal validation was performed to estimate the shrinkage factor and adjust for any overestimation of the area under the receiver operating characteristic (ROC) curve (AuROC).

Model performance was evaluated by discrimination and calibration. Discrimination was assessed using the AuROC, whereas calibration was examined with the Hosmer‒Lemeshow test and by comparing predicted risk probabilities with actual observed probabilities (calibration curve). A predictive scoring model was then developed based on the final multivariable logistic regression coefficients, incorporating the adjusted bootstrap internal validation.

Finally, an ROC curve was generated to determine the optimal cutoff point for predicting pre-resection hydrocephalus. Sensitivity, specificity, positive predictive value, negative predictive value, positive likelihood ratio, negative likelihood ratio, and the AuROC were reported.

## Results

### Patient characteristics

A total of 421 patients were diagnosed with posterior cranial fossa tumors based on initial cranial imaging. Among them, 160 (38%) presented with hydrocephalus, while 261 (62%) did not. The median age was 53 years (range 19‒87). Males comprised 133 (31.6%) of the cohort, and 288 (68.4%) were female.

### Clinical presentation

The most frequent presenting symptom was cranial nerve dysfunction (71.7%), followed by ataxia (60.8%), IICP symptom (26.8%), cognitive impairment (9%), and deterioration of consciousness (4.7%).

### Tumor pathology

Schwannoma was the most common tumor (45.3%). Malignant tumors (21%) and meningiomas (20.5%) were the next most prevalent, occurring at similar frequencies.

### Radiographic findings

Mean Evans’ indices were 0.33 ± 0.04 in the hydrocephalus group and 0.25 ± 0.02 in the non-hydrocephalus group, with an overall mean of 0.28 ± 0.05. Most tumors (75.1%) involved or compressed the cerebellar cortex. On post-contrast MRI, 97.9% of tumors showed contrast enhancement, and 66.8% of all tumors demonstrated heterogeneous enhancement.

### Univariate analysis

Table 1 summarizes the univariate associations between each variable and the presence of hydrocephalus.

**Table 1:** Univariate analysis of factors associated with pre-resection hydrocephalus

Variables	Overall (n = 421)	Comparison between the groups with and without hydrocephalus		
		Hydrocephalus (n = 160)	No hydrocephalus (n = 261)	*p* value
Age (years), median (range)	53 (19–87)	53.5 (22–81)	53 (19–87)	0.567
Male, n (%)	133 (31.6)	61 (38.1)	72 (27.9)	0.024^a^
Duration of presenting symptom (month), median (range)	6 (0.03–240)	3 (0.03–240)	8 (0.03–156)	< 0.001^a^
Presenting symptom, n (%)				
IICP symptom	113 (26.8)	84 (52.5)	29 (11.1)	< 0.001^a^
Ataxia	256 (60.8)	127 (79.3)	129 (49.4)	< 0.001^a^
Cognitive impairment	38 (9.0)	32 (20.0)	6 (2.2)	< 0.001^a^
Deterioration of consciousness	20 (4.7)	19 (11.8)	1 (0.3)	< 0.001^a^
Cranial nerve dysfunction	302 (71.7)	93 (58.1)	209 (80.0)	< 0.001^a^
Tumor pathology, n (%)				
Malignant	88 (21.0)	60 (37.5)	28 (10.8)	< 0.001^a^
Schwannoma	191 (45.3)	53 (33.1)	138 (52.8)	< 0.001^a^
Meningioma	86 (20.5)	25 (15.6)	61 (23.3)	0.056
Glial	15 (3.5)	8 (5.0)	7 (2.7)	0.213
Tumor volume (cm^3^), mean ± SD	15.5±16.6	25.4±20.4	9.53±9.85	< 0.001^a^
Posterior cranial fossa volume (cm^3^), mean ± SD	173.7±24	174.9±27.7	172.9±21.51	0.388
Clival angle (degree), mean ± SD	122.6±7.05	123.4±7.06	122.1±7.02	0.889
Straight sinus angle (degree), mean ± SD	42.3±8.37	42.2±8.88	42.4±8.55	0.890
Intra-axial location, n (%)	129 (30.6)	78 (48.8)	51 (19.5)	< 0.001^a^
Midline location, n (%)	125 (29.7)	58 (36.2)	67 (25.6)	0.021^a^
Cerebellar cortex involvement, n (%)	316 (75.1)	110 (68.8)	206 (79.0)	0.019^a^
Brainstem involvement, n (%)	52 (12.4)	18 (11.3)	34 (13.1)	0.590
Tumor enhancement, n (%)	412 (97.9)	158 (98.8)	254 (97.4)	0.493
Heterogeneous enhancement, n (%)	272 (66.8)	123 (78.2)	149 (59.8)	< 0.001^a^
Peritumoral vasogenic edema, n (%)	158 (37.5)	115 (71.9)	43 (16.5)	< 0.001^a^
Intratumoral cyst, n (%)	122 (29.0)	54 (33.8)	68 (26.1)	0.091

*CI* confidence interval, *IICP* increased intracranial pressure, *n* number of patients *OR* odds ratio, *SD* standard deviation

^a^ *p* value < 0.05 indicates a statistically significant difference

- *Demographic factors*: Male sex, shorter duration of presenting symptoms, IICP symptom, ataxia, cognitive impairment, deterioration of consciousness, and malignancy were positively correlated with hydrocephalus. In contrast, cranial nerve dysfunction and schwannoma were inversely associated. Meningioma showed a trend toward being protective, but did not reach statistical significance (*p* = 0.056).
- *Radiographic factors*: Larger tumor volume, intra-axial (brain parenchymal) location, midline position, heterogeneous contrast enhancement, and peritumoral vasogenic edema were significantly associated with hydrocephalus. Conversely, tumor involvement or compression of the cerebellar cortex was the only radiographic variable significantly protective against hydrocephalus (*p* = 0.019, OR 0.59, 95% CI 0.38‒0.92).
- *Craniometric variables*: Posterior cranial fossa volume, as well as the clival and straight sinus angles, did not exhibit significant associations with the development of hydrocephalus.

### Strength of association

Table 2 summarizes the strength of association between individual factors and the occurrence of hydrocephalus. Notably, deterioration of consciousness showed a markedly high OR (35.04, 95% CI 4.64‒264.46). Other factors with strong associations were peritumoral vasogenic edema (OR 12.96, 95% CI 8.06‒20.84), cognitive impairment (OR 10.63, 95% CI 4.33‒26.07), IICP symptom (OR 8.84, 95% CI 5.39‒14.51), malignancy (OR 4.99, 95% CI 3.01‒8.28), and ataxia (OR 3.94, 95% CI 2.50‒6.20).

**Table 2:** Strength of association between variables and pre-resection hydrocephalus

Variables		OR	(95% CI)	*p* value
Age		0.99	(0.98–1.00)	0.116
Male		1.62	(1.06–2.46)	0.025^a^
Duration of presenting symptoms		0.98	(0.97–0.99)	0.044^a^
Presenting symptoms				
IICP symptom		8.84	(5.39–14.51)	< 0.001^a^
Ataxia		3.94	(2.50–6.20)	< 0.001^a^
Cognitive impairment		10.63	(4.33–26.07)	< 0.001^a^
Deterioration of consciousness		35.04	(4.64–264.46)	< 0.001^a^
Cranial nerve dysfunction		0.35	(0.22–0.53)	< 0.001^a^
Tumor pathology				
Malignant		4.99	(3.01–8.28)	< 0.001^a^
Schwannoma		0.44	(0.29–0.66)	< 0.001^a^
Meningioma		0.61	(0.36–1.02)	0.057
Glial		1.91	(0.68–5.37)	0.220
Tumor volume		1.10	(1.08–1.13)	< 0.001^a^
Posterior cranial fossa volume		1.00	(0.99–1.01)	0.416
Clival angle		1.03	(0.99–1.06)	0.790
Straight sinus angle		0.99	(0.98–1.02)	0.889
Intra-axial location		3.92	(2.53–6.05)	< 0.001^a^
Midline location		1.65	(1.08–2.52)	0.022^a^
Cerebellar cortex involvement		0.59	(0.38–0.92)	0.020^a^
Brainstem involvement		0.85	(0.46–1.56)	0.591
Tumor enhancement		2.18	(0.45–10.61)	0.336
Heterogeneous enhancement		2.48	(1.58–3.89)	< 0.001^a^
Peritumoral vasogenic edema		12.96	(8.06–20.84)	< 0.001^a^
Intratumoral cyst		1.45	(0.94–2.22)	0.092

*CI* confidence interval, *IICP* increased intracranial pressure, *OR* odds ratio

^a^ *p* value < 0.05 indicates a statistically significant difference

### Multivariable analysis

**Fig. 3:**
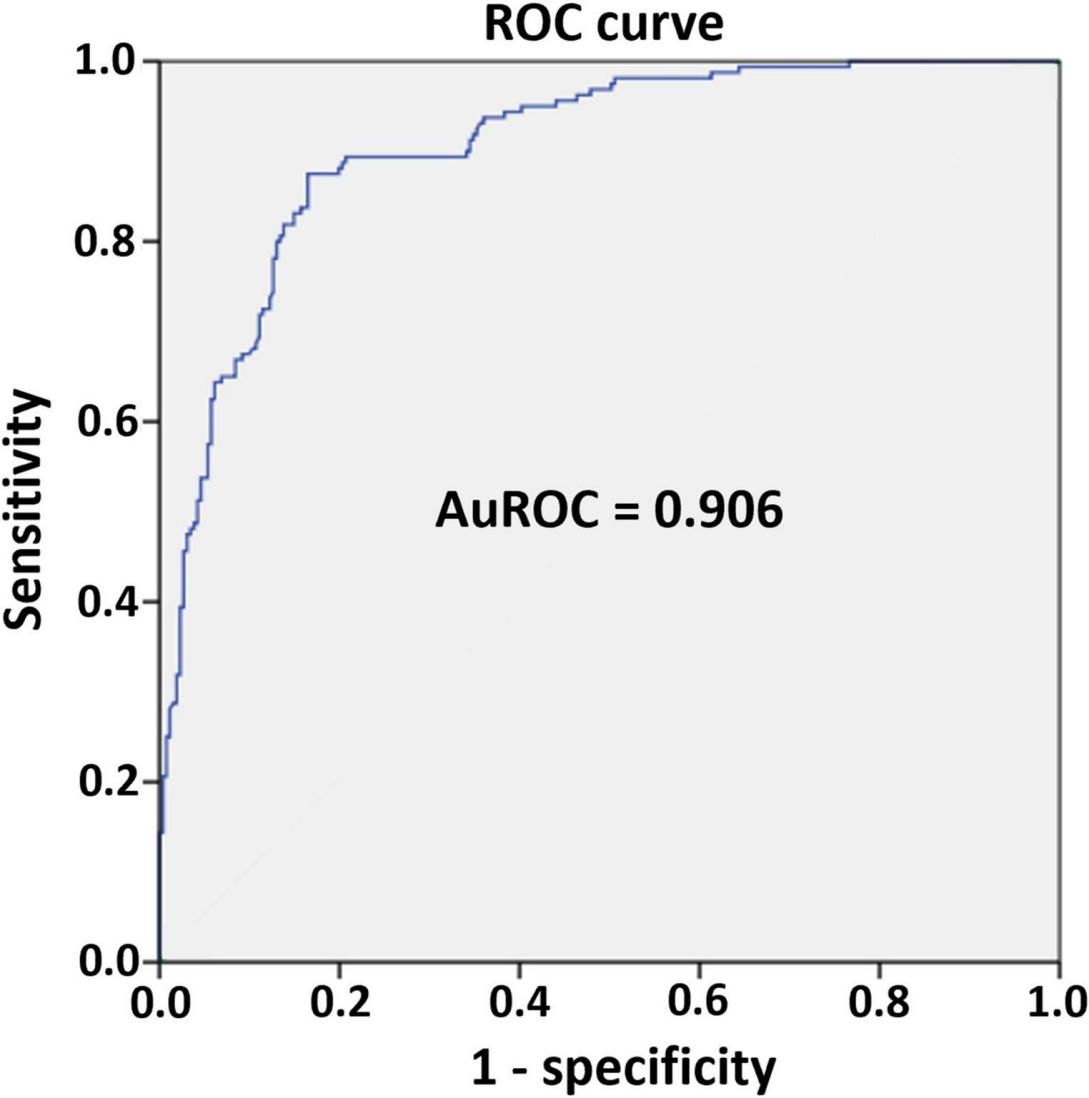
Receiver operating characteristic (ROC) curve of binary logistic regression. The ROC curve demonstrates the model’s predictive performance, with an area under the ROC curve (AuROC) of 0.906 (95% confidence interval: 0.878–0.934)

All variables with a *p* value less than 0.2 in univariate testing were entered into the multivariable model. These were age, sex, duration of presenting symptoms, IICP symptom, ataxia, cognitive impairment, deterioration of consciousness, cranial nerve dysfunction, malignancy, schwannoma, meningioma, tumor volume, intra-axial tumor location, midline location, cerebellar cortex involvement, heterogeneous tumor enhancement, peritumoral vasogenic edema, and intratumoral cystic appearance. After collinearity testing and backward (conditional) logistic regression, seven factors remained in the final model: age, IICP symptom, ataxia, cognitive impairment, deterioration of consciousness, tumor volume, and peritumoral vasogenic edema. Of these seven, IICP symptom, ataxia, cognitive impairment, tumor volume, and peritumoral vasogenic edema were significantly associated with hydrocephalus. Age (*p* = 0.081) and deterioration of consciousness (*p* = 0.050) did not reach statistical significance. The final multivariable findings are shown in Table 3. Figure 3 illustrates the ROC curve, with an AuROC of 0.906 (95% CI 0.878‒0.934).

**Table 3:** Multivariable analysis of factors associated with pre-resection hydrocephalus

Variable		Crude OR (95% CI)	*p* value	Adjusted OR (95% CI)	*p* value
Age		0.99 (0.98–1.00)	0.116	1.03 (0.99–1.04)	0.081
IICP symptom					
Absent		1.00		1.00	
Present		8.84 (5.39–14.51)	< 0.001^a^	4.08 (2.08–8.02)	< 0.001^a^
Ataxia					
Absent		1.00		1.00	
Present		3.94 (2.50–6.20)	< 0.001^a^	2.49 (1.23–5.04)	0.011^a^
Cognitive impairment					
Absent		1.00		1.00	
Present		10.63 (4.33–26.07)	< 0.001^a^	6.92 (2.17–22.01)	0.001^a^
Deterioration of consciousness					
Absent		1.00		1.00	
Present		35.04 (4.64–264.46)	< 0.001^a^	12.79 (0.99–164.40)	0.050
Tumor volume		1.10 (1.08–1.13)	< 0.001^a^	1.07 (1.04–1.11)	< 0.001^a^
Peritumoral vasogenic edema					
Absent		1.00		1.00	
Present		12.96 (8.06–20.84)	< 0.001^a^	6.38 (3.52–11.56)	< 0.001^a^

*CI* confidence interval, *IICP* increased intracranial pressure, *OR* odds ratio

^a^ *p* value < 0.05 indicates a statistically significant difference

### Predictive scoring model

A predictive scoring model was developed based on the five significant variables in the final logistic regression (four categorical factors plus tumor volume as a quantitative factor). These variables and scores were described as the follows.

- Cognitive impairment: absent = 0, present = 4.
- Peritumoral vasogenic edema: absent = 0, present = 3.
- IICP symptom: absent = 0, present = 2.
- Ataxia: absent = 0, present = 1.
- Tumor volume: the score was calculated by tumor volume (cm^3^)/10 × 1.

Total score was summation of all scores, and could be expressed in decimal number.

Table 4 shows the scoring system, which uses an optimal cutoff score of 4 to distinguish high from low probabilities of pre-resection hydrocephalus. At this cutoff, the model demonstrated an AuROC of 0.84, with 87.5% sensitivity, 81.2% specificity, a positive predictive value of 74.1, a negative predictive value of 91.4, a positive likelihood ratio of 4.66, and a negative likelihood ratio of 0.15 (Supplementary Table).

**Table 4:** Predictive scoring model for pre-resection hydrocephalus

Variable		Score
Cognitive impairment		
Absent		0
Present		4
Peritumoral vasogenic edema		
Absent		0
Present		3
IICP symptom		
Absent		0
Present		2
Ataxia		
Absent		0
Present		1
Tumor volume (cm^3^)		Volume (cm^3^)/10 × 1

*IICP* increased intracranial pressure

Total predictive score ≥ 4 indicates high probability in occurrence of hydrocephalus

Total score can be expressed in a decimal number

## Discussion

Hydrocephalus is common in patients with posterior cranial fossa tumors, especially in pediatric populations. Among children with these tumors, hydrocephalus occurs in about 73%‒100% of cases [14, 44–46], compared with about 30%‒40% in adults [47–49]. Posterior fossa tumors frequently obstruct CSF flow [15]. The primary mechanism involves direct compression of CSF pathways, causing obstructive hydrocephalus [21, 50–52]. Hemodynamic disturbances, such as increased vascular resistance in intracranial venous sinuses, also contribute [53]. Both intra-axial and extra-axial lesions can compress major venous sinuses and impair venous outflow, leading to hydrocephalus [54]. Although large posterior fossa tumors commonly induce hydrocephalus, some small- or medium-sized tumors also produce it. Hence, factors beyond tumor size may be relevant.

In our univariate analysis, male sex showed a higher risk of hydrocephalus (OR 1.62, *p* = 0.025), consistent with prior studies [17, 55]. Typical neurologic symptoms, including IICP symptom, ataxia, cognitive impairment, and altered consciousness, were also associated. These findings align with the effects of large tumor volume, vasogenic edema, and hydrocephalus itself [56]. Interestingly, cranial nerve dysfunction was more frequent in patients without hydrocephalus (OR 0.35, *p* < 0.001). Patients presenting with isolated cranial neuropathies, such as hearing loss or facial numbness, often seek early medical attention, facilitating diagnosis before obstructive hydrocephalus develops. A shorter presenting symptom duration (median 3 months) was linked with hydrocephalus (*p* = 0.044), whereas longer, more tolerable symptom courses (median 8 months) carried lower risk. Rapidly growing tumors or significant vasogenic edema, often seen in malignant neoplasms, may overwhelm intracranial compensatory mechanisms and thus cause obstructive hydrocephalus.

Malignant posterior cranial fossa tumors were strongly associated with hydrocephalus (OR 4.99, *p* < 0.001). Rapid tumor growth and vasogenic brain edema narrow the posterior cranial fossa, ultimately resulting in obstructive hydrocephalus. In one study of hydrocephalus management following posterior fossa tumor resection, metastasis was the most frequent malignant tumor causing preoperative hydrocephalus in adults (29.5%), whereas medulloblastoma was predominant in children [47]. By contrast, benign tumors showed a different pattern. Schwannomas were found significantly more often in patients without hydrocephalus (OR 0.44, *p* < 0.001), and meningiomas showed a similar, though not statistically significant, trend (OR 0.61, *p* = 0.057). Schwannomas and meningiomas grow slowly and gradually compress the brain or cranial nerves [57, 58]. This protracted progression likely allows for intracranial hydrodynamic compensation, reducing the likelihood of hydrocephalus, even with large tumors. Moreover, patients with benign tumors often experience cranial neuropathy (such as hearing loss or facial paresthesia), prompting earlier medical attention before obstructive hydrocephalus develops.

Prior to this study, we hypothesized that steep clival and straight sinus angles might predispose individuals to hydrocephalus by reducing posterior cranial fossa volume. However, none of the craniometric variables (posterior fossa volume, clival angle, and straight sinus angle) was significantly associated with hydrocephalus. Instead, tumor volume emerged as a strong predictor in both univariate and multivariable analyses. These findings align with earlier reports showing that larger tumor volume heightens the risk of hydrocephalus before resection or radiosurgery [59–61].

Tumors located in the midline or deep regions often lead to hydrocephalus by obstructing the ventricular system, particularly the fourth ventricle. Patients with intrinsic brainstem tumors frequently develop multiple cranial nerve deficits before hydrocephalus becomes apparent. Previous studies similarly reported that midline tumors or those compressing or infiltrating the fourth ventricle are associated with persistent hydrocephalus [61–65]. Intra-axial tumors in the posterior fossa tend to be malignant (e.g., metastases and malignant gliomas), which predispose to hydrocephalus. A study by Shabo et al. of 130 patients with metastatic posterior cranial fossa tumors found that all required preoperative external ventricular drainage, highlighting the high prevalence of obstructive hydrocephalus in metastatic disease [65].

Heterogeneous tumor enhancement commonly reflects malignancy or higher tumor aggressiveness, such as metastatic lesions, malignant gliomas, medulloblastomas, ependymomas, or medium-to-large vestibular schwannomas with cystic components. These tumors frequently lead to hydrocephalus, whereas those with homogeneous enhancement, like meningiomas or very small schwannomas, are less likely to cause it. Peritumoral vasogenic brain edema was also a robust predictor of hydrocephalus in this study. It is often seen in aggressive tumors, such as glioblastomas and metastatic lesions [66–68], which readily produce hydrocephalus if located in the posterior fossa. Although intratumoral cystic changes can accelerate tumor expansion, thereby promoting hydrocephalus, they did not reach statistical significance in our univariate or multivariable analyses. Similarly, Shin et al. reported that vestibular schwannomas with large cystic components were associated with postoperative hydrocephalus [69].

In the multivariate analysis, IICP symptom, ataxia, cognitive impairment, greater tumor volume, and peritumoral vasogenic edema had significant associations with pre-resection hydrocephalus. These variables emerged as the most important predictors and were thus incorporated into the scoring model. The proposed predictive score aimed to identify patients with a great risk of hydrocephalus development before tumor resection. It may help neurosurgeons with decision-making of early surgical interventions. In patients who are at high risk, their operative schedules can be brought forward to prevent the detrimental effects of hydrocephalus.

Table 5 [9, 14, 21, 44–49, 59–65, 67, 69–103] provides a comprehensive literature review of hydrocephalus in posterior cranial fossa tumors. Most articles examined post-resection hydrocephalus or the need for CSF diversion, whereas publications focusing on pre-resection hydrocephalus were scarce. Based on this review, Table 6 [6, 14, 21, 45–49, 59–65, 67, 69, 71, 73, 75–77, 80, 81, 83–85, 87, 89–99, 101, 103] summarizes the risk factors or predictors of hydrocephalus and CSF diversion requirements, stratified by variable type, treatment phase, and patient age group.

**Table 5:** Summary of studies on hydrocephalus in posterior cranial fossa tumors [9, 14, 21, 44–49, 59–65, 67, 69–103]

Authors (year) [reference]	Investigation	Pre-resection permanent CSF diversion	Tumor pathology	*n*	Age (range)	Prevalence of hydrocephalus	Risk factors/predictors of hydrocephalus or requirement of CSF diversion
Schmid and Seiler (1986) [44]	Post-resection VPS	None	Adult: medulloblastoma 2.6%, astrocytoma 7.9%, ependymoma 10.5%, hemangioblastoma 26.3%, other neuroepithelial tumors 31.6%, non-neuroepithelial tumor 21.1% Pediatric: medulloblastoma 43.5%, astrocytoma 30.4%, ependymoma 17.3%, other neuroepithelial tumors 4.4%, non-neuroepithelial tumor 4.4%	61 (adult 38, pediatric 23)	NR	Preop: adult 38/38 (100%), pediatric 23/23 (100%) Postop VPS: adult 0/38 (0%), pediatric 4/23 (17.4%)	No variables were associated with post-resection requirement of VPS
Dias and Albright (1989) [45]	Post-resection VPS	Preop VPS 25/58 (43.1%)	NR	58	Childhood, no age reported	Preop 58/58 (100%) Postop VPS 9/58 (15.5%)	Incomplete tumor resection, the dura left open following tumor resection
Briggs et al. (1993) [70]	Post-resection VPS	Preop VPS 2/43 (4.7%)	Vestibular schwannoma 100%	43	Mean 53.5 y (R 19–71)	Preop 43/43 (100%) Postop VPS 2/42 (4.7%)	NR
Culley et al. (1994) [21]	Post-resection VPS	NR	Medulloblastoma 44%, astrocytoma 40%, ependymoma 14%, other 3%	117	R 4–201 m	Preop 97/117 (82.9%) Postop VPS 42/117 (35.9%)	Young age, midline tumor location, subtotal tumor resection, prolonged EVD placement, cadaveric dural grafts, pseudomeningocele, CSF infections
Lee et al. (1994) [71]	Post-resection shunt	Excluded	Medulloblastoma 100%	42	Mean 8.1 ± 5.3 y	Preop NR Postop shunt 17/42 (40.5%)	Young age, severity of preop hydrocephalus (frontal horn ratio, ventricular body ratio), more extensive tumors (Chang’s stage T3 and T4)
Atlas et al. (1996) [59]	Pre-resection hydrocephalus	Preop VPS 2/104 (1.9%)	Vestibular schwannoma 100%	104	Mean 53 y (R 19–87)	Preop 14/104 (13.5%) Postop 0/104 (0%)	Large tumor size
Kumar et al. (1996) [46]	Post-resection VPS	None	Medulloblastoma 50.3%, astrocytoma 37.1%, ependymoma 10.3%, other 2.3%	175	R 4 m–14 y 6 m	Preop 165/175 (94.3%) Postop VPS 33/175 (18.9%)	Young age, tumor pathology (ependymoma and medulloblastoma), incomplete tumor resection
Imieliński et al. (1998) [72]	Post-resection VPS	Preop VPS 47/95 (49.5%)	Astrocytoma 33.7%, medulloblastoma 22.1%, ependymoma 16.8%, brainstem tumor 13.7%, other 13.7%	95	Mean 8.2 y	Preop 75/95 (78.9%) Postop VPS 12/95 (12.6%)	NR
Kazan et al. (1998) [73]	Post-resection VPS	None	Medulloblastoma 39.3%, astrocytoma 35.7%, other 25%	28	Mean 9.8 ± 3.9 y	Preop NR Postop VPS 8/28 (28.6%)	Young age, incomplete tumor resection, prolonged EVD placement, high postop ICP levels (EVD dependent group)
Sainte-Rose et al. (2001) [74]	Post-resection hydrocephalus and permanent CSF diversion	Preop ETV 67/196 (34.2%)	Medulloblastoma 50%, ependymoma 26.5%, astrocytoma 52%, ganglioglioma 2.6%, PNET 5.6%, other 18.4%	196	Mean 7.1 y (R 46 d–16 y)	Preop 149/196 (76%) Postop 26/196 (13.3%) (VPS 20/26, ETV 6/26)	NR
Bognár et al. (2003) [75]	Post-resection hydrocephalus and shunt	Excluded	Astrocytoma 41%, medulloblastoma 39%, ependymoma 11%, other 8%	180	Mean 7.4 y (R 3 m–16 y)	Preop 137/180 (76.1%) Postop shunt 16/180 (8.9%)	Young age, ependymoma, preop or postop EVD insertion
Gnanalingham et al. 2003 [76]	Post-resection permanent CSF diversion	Preop VPS 5/89 (5.6%), ETV 2/89 (2.2%)	Astrocytoma 51.7%, medulloblastoma 23.6%, ependymoma 13.5%, other 11.2%	89	Mean 5.9 ± 0.4 y (R 0.02–15)	Preop 15/89 (16.9%) Postop permanent CSF diversion 11/89 (12.4%) (VPS 10/11, ETV 1/11)	Midline tumor location, preop and intraop CSF drainage, longer hospital stay, other tumor pathology (pineoblastoma, pineocytoma, germ cell tumors, meningioma)
Tanaka et al. (2003) [60]	Pre-resection hydrocephalus	Preop VPS 8/236 (3.4%)	Vestibular schwannoma 100%	236	Mean 53.6 ± 13.2 y (R 17–83)	Preop 33/236 (14%) Intraop VPS 1/236 (0.42%) Postop VPS 7/236 (3%)	Older age, large tumor size
Morelli et al. (2005) [77]	Post-resection hydrocephalus and permanent CSF diversion	Preop VPS 7/160 (4.4%)	Pilocytic astrocytoma 25.6%, ependymoma 19.4%, medulloblastoma 16.9%, diffuse astrocytoma 14.4%, anaplastic astrocytoma 5%, papilloma 3.1%, other 5.6%	160	< 1y: 16/160 (10%) 1–3 y: 64/160 (40%) 3–10 y: 62/160 (38.8%) > 10 y: 18/160 (11.2%)	Preop 114/160 (71.3%) Postop permanent CSF diversion 17/160 (10.6%) (VPS 5/17, ETV 8/17, asymptomatic and follow-up 4/17)	Severe preop hydrocephalus, medulloblastoma
Rogg et al. (2005) [61]	Pre-radiosurgery hydrocephalus	NR	Vestibular schwannoma 100%	157	Mean 55 y (R 19–93)	Pre-radiosurgery 28/157 (17.8%)	Tumor volume, severity of fourth ventricular compression
Abdollahzadeh-Hosseini et al. (2006) [78]	Post-resection VPS	Preop shunting 81/108 (75%) (VPS 77/81, VAS 4/81)	Astrocytoma 44.4%, medulloblastoma 26.9%, brainstem glioma 13%, ependymoma 11.1%, other 4.6%	108	Mean 8.9 ± 4.4 y (R 3 m–18 y)	Preop 99/108 (91.7%) Postop VPS 3/108 (2.8%)	NR Complication rate (CSF leakage, septic meningitis) was lower in patients with pre-resection shunting
Due-Tønnessen and Helseth (2007) [79]	Post-resection hydrocephalus and permanent CSF diversion	Preop ETV 2/87 (2.3%)	Medulloblastoma 40.2%, astrocytoma 43.7%, ependymoma 16.1%	87	Mean 7.3 y (R 0.2–19.7)	Preop 69/87 (79.3%) Postop permanent CSF diversion (VPS, ETV) 28/69 (40.6%) 41/69 (59.4%) with preop hydrocephalus had cured hydrocephalus by tumor resection alone	NR Cure rate of hydrocephalus: astrocytoma 83%, ependymoma 54%, medulloblastoma 47%
Fukuda et al. (2007) [80]	Pre-resection hydrocephalus	Preop VPS 4/68 (5.9%)	Vestibular schwannoma 100%	68	Mean 51.4 y (R 19–76)	Preop 16/68 (2.4%) Postop VPS 2/68 (2.9%)	High CSF protein concentration
Santos de Oliveira et al. (2008) [81]	Post-resection hydrocephalus and permanent CSF diversion	None	Medulloblastoma 40.6%, pilocytic astrocytoma 32.8%, ependymoma 7.8%, ganglioglioma 6.3%, other 12.5%	64	Mean 9 y 2 m (R 3 m–18 y)	Preop 56/64 (87.5%) Postop permanent CSF diversion 22/64 (34.4%) (VPS 19/22, ETV 3/22)	Young age, severity of preop hydrocephalus (ventricular index), midline tumor location, ependymoma
Tamburrini et al. (2008) [82]	Post-resection ETV	NR	Tumor pathology was reported only in patients underwent post-resection ETV (*n* = 30): medulloblastoma 33.3%, pilocytic astrocytoma 40%, ependymoma 26.7%	104	Age was reported only in patients underwent post-resection ETV (*n* = 30) Mean 6.8 ± 3.8 y (R 1–15)	Preop 104/104 (100%) (moderate 15/104, severe 89/104) Postop ETV 30/104 (28.8%)	NR
Riva-Cambrin et al. (2009) [83]	Post-resection hydrocephalus and shunt	Excluded	Preop predicted tumor diagnosis on imaging: pilocytic astrocytoma 33.2%, medulloblastoma 22.4%, ependymoma 30.8%, dorsally exophytic brainstem glioma 6.6%, other 6.9%	343	Mean 84 ± 52.1 m	Preop 279/343 (81.3%) (mild 99/343, moderate 178/343, severe 2/343) Postop shunt 107/343 (31.2%)	The Canadian Preoperative Prediction Rule for Hydrocephalus (CPPRH): age < 2 y (score of 3), cerebral metastasis (score of 3), moderate to severe hydrocephalus (score of 2), papilledema (score of 1), preop predicted tumor diagnosis of ependymoma, medulloblastoma or dorsally exophytic brainstem glioma on imaging (score of 1) Patients with total scores ≥ 5 were classified as the high-risk group
Gopalakrishnan et al. (2012) [84]	Post-resection hydrocephalus and permanent CSF diversion	Excluded	Medulloblastoma 45.2%, astrocytoma 39.2%, ependymoma 9.6%, other 6%	84	Mean 8 y (R 1.5–18)	Preop 80/84 (95.2%) Postop permanent CSF diversion 25/84 (29.8%) (VPS 15/25, ETV 15/25)	Duration of symptom < 3 m, severity of hydrocephalus (Evan’s ratio > 0.33, FOHR > 0.46), midline tumor location, medulloblastoma and ependymoma, EVD insertion, postop pseudomeningocele or meningitis
Foreman et al. (2013) [14]	Post-resection hydrocephalus and shunt	Excluded	Preop predicted tumor diagnosis on imaging: pilocytic astrocytoma 36.8%, medulloblastoma 26.3%, ependymoma 22.4%, dorsally exophytic brainstem glioma 4%, other 10.5%	76	Mean 92.4 ± 56.1 m	Preop 39/76 (51.3%) Postop shunt 16/76 (21.1%)	Age < 2 y, moderate to severe hydrocephalus, preop predicted tumor diagnosis of ependymoma, medulloblastoma or dorsally exophytic brainstem glioma on imaging, transependymal edema
Aljubour et al. (2017) [85]	Post-resection VPS	Excluded	Medulloblastoma 65.4%, astrocytoma 23.1%, ependymoma 11.5%	52	< 3 y: 10/52 (19.2%) > 3 y: 42/52 (80.8%)	Preop NR Postop VPS 24/52 (46.2%)	Age < 3 y, subtotal tumor resection
Ghani et al. (2017) [86]	Post-resection VPS	None	Medulloblastoma 57.9%, pilocytic astrocytoma 21.1%, anaplastic ependymoma 13.2%, other 7.8%	38	Mean 61.5 ± 30.4 m	Preop 38/38 (100%) Postop VPS 9/38 (23.7%)	Preop duration of EVD was not associated with requirement of postop VPS
Chel’diev et al. (2018) [87]	Post-resection VPS	None	Pilocytic astrocytoma 36.8%, medulloblastoma 35.5%, anaplastic ependymoma 14.8%, other 12.9%	155	Mean 6.4 ± 4.2 y (R 6–18)	Preop NR Postop VPS 13/155 (8.4%)	Anaplastic ependymoma
Hamdan and Abd El-Hakeem (2018) [9]	Post-resection hydrocephalus	Preop VPS 3/30 (10%)	Intra-axial posterior cranial fossa tumors: medulloblastoma 40%, pilocytic astrocytoma 33.3%, anaplastic astrocytoma 13.3%, metastatic adenocarcinoma 6.7%, anaplastic ependymoma 3.33%, hemangioblastoma 3.33%	30	Mean 19.4 ± 18.2 y (R 11 m–59 y)	Preop 12/30 (40%) Postop 6/30 (20%)	NR
Marx et al. (2018) [88]	Post-resection hydrocephalus	Preop ETV 11/243 (4.5%)	Vestibular schwannoma 32.5%, metastasis 28%, meningioma 21%, glioma 7%, other 11.5%	243	Mean 54.1 y (R 20–87)	Preop 52/243 (21.4%) Postop 7/243 (2.9%)	NR
Abraham et al. (2019) [89]	Post-resection CSF diversion	Excluded	Medulloblastoma 51.3%, pilocytic astrocytoma 35.8%, ependymoma 8.8%, high-grade glioma 2.4%, choroid plexus papilloma 1.4%	148	Mean 8.7 y (R 1–17)	Preop 131/148 (89%) Postop CSF diversion 14/148 (9.4%) (VPS 12/14, EVD 1/14, thecoperitoneal shunt 1/14)	Age < 6 y, presence of intraventricular blood on postop cranial CT
Helmbold et al. (2019) [90]	Post-resection hydrocephalus and VPS	Preop ETV 1/70 (1.4%)	Medulloblastoma 40%, astrocytoma 31.4%, ependymoma 21.4%, other 7.2%	70	Median 8.2 y (R 0.4–20.8)	Preop 51/70 (72.9%) Postop hydrocephalus 45/70 (64.3%) (VPS 15/70)	Age < 3 y, periop EVD placement, signs of hydrocephalus in postop imaging, a FOHR > 0.46 within the first 72 h postoperatively, presence of intraventricular blood postoperatively
Araki et al. (2020) [91]	Post-resection VPS	None	Medulloblastoma 35.8%, pilocytic astrocytoma 50%, diffuse astrocytoma 7.1%, hemangioblastoma 7.1%	14	Median 8 y (R 0–15)	Preop 12/14 (85.7%) Postop VPS 2/14 (14.3%)	Age < 2 y, medulloblastoma, tumor dissemination, partial tumor resection
Bhuyan et al. (2020) [92]	Post-resection VPS	None	Medulloblastoma 65.4%, ependymoma 23.1%, astrocytoma 11.5%	26	Mean 8.6 ± 4.6 y	Preop mean Evan's ratio 0.45, 26/26 (100%) had periventricular lucency, no prevalence of hydrocephalus reported Postop VPS 7/26 (26.9%)	Younger age, incomplete tumor resection, longer period of artificial ventilatory support, EVD insertion, postop duration of EVD insertion
Chen et al. (2020) [93]	Post-resection hydrocephalus and CSF diversion	NR	Fourth ventricular tumors: ependymoma 30.6%, medulloblastoma 24.2%, pilocytic astrocytoma 16.5%, other 28.7%	121	Median 25 y (IQR 9–41)	Preop 70/121 (57.9%) Postop CSF diversion 15/121 (12.4%) (VPS 10/15, 5/15 EVD)	All patients: superior tumor extension (into the aqueduct), incomplete tumor resection Subgroup of periop EVD placement: superior tumor extension (into the aqueduct), incomplete tumor resection
Srinivasan et al. (2020) [94]	Post-resection VPS	Preop ETV 28/95 (29.5%)	Medulloblastoma 32.6%, pilocytic astrocytoma 31.6%, ependymoma 21.1%, other 14.7%	95	Median 7 y (IQR 7)	Preop mean FOHR 0.42, no prevalence of hydrocephalus reported Postop VPS 17/95 (17.9%)	Ependymoma, high modified Canadian Preoperative Prediction Rule for Hydrocephalus (mCPPRH) score
Won et al. (2020) [47]	Post-resection hydrocephalus	Excluded	Pediatric: medulloblastoma 34%, astrocytoma 24.4%, pilocytic astrocytoma 22%, other 19.6% Adult: metastasis 29.5%, meningioma 22%, vestibular schwannoma 17.8%, other 30.7%	302 (pediatric 40, adult 262)	Pediatric: median 12 y (IQR 7–14) Adult: median 55 y (IQR 41–64)	Preop: pediatric 22/40 (55%), adult 100/262 (38.2%) Postop: pediatric 15/40 (37.5%), adult 18/262 (6.9%)	Pediatric: age ≤ 2 y, medulloblastoma, brainstem compression Adult: pilocytic astrocytoma, preop hydrocephalus
Won et al. (2020) [67]	Post-resection VPS	None	Intraparenchymal tumor: metastasis 60.2%, hemangioblastoma 16.3%, vascular malformations (AVM, cavernoma 8.2%, other 6.1% Extraparenchymal tumor: meningioma 45.5%, vestibular schwannoma 38.4%, fourth ventricle ependymoma 16.2%	197	Mean 53 ± 15.1 y	Preop 68/197 (34.5%) Postop VPS 14/197 (7.1%)	Intraparenchymal tumor - Risk factors: preop hydrocephalus, periventricular CSF capping - Protective factors: semi-sitting surgical position, total tumor resection Extraparenchymal tumor - Risk factors: petroclival or midline tumor location, perilesional edema, preop hydrocephalus
Pitsika et al. (2021) [95]	Post-resection VPS	Excluded	Medulloblastoma 38.7%, ependymoma 22.7%, astrocytoma 24%, dorsally exophytic pontine glioma 1.3%, other 13.3%	75	Mean 7 y (R 0.1–18)	Preop 55/75 (73.3%) Postop VPS 8/75 (10.7%)	EVD insertion
Saad et al. (2021) [48]	Post-resection VPS	None	The most common was metastasis, followed by meningioma, schwannoma, hemangioblastoma, and other, respectively	617	Mean 51 ± 16 y	Preop 178/587 (30%) Postop VPS 81/617 (13.1%)	Using preop variables: presence of preop transependymal flow, preop tumor size Using preop and postop variables: presence of preop transependymal flow, preop or intraop EVD insertion, presence of postop IVH, surgical complications, failed gross total resection, intra-axial tumor location Intra-axial tumor group: presence of preop transependymal flow, presence of postop IVH, surgical complications, failed gross total resection Extra-axial tumor group: surgical complications
Shin et al. (2021) [69]	Post-resection persistent hydrocephalus (treatment failure)	Preop ETV 57/122 (46.7%) Excluded preop VPS	Vestibular schwannomas treated by tumor resection alone 60/122 (49.2%), tumor resection with ETV 57/122 (46.7%), tumor resection with EVD 5/122 (4.1%)	122	Mean 53.1 y (R 19–80)	Preop 122/122 (100%) Postop persistent hydrocephalus (treatment failure) 14/122 (11.5%) Treatment failure rate: tumor resection alone 5/60 (8.3%), tumor resection with ETV 7/57 (12.3%), tumor resection with EVD 2/5 (40%)	Severe preop hydrocephalus (Evans’ index > 0.4), cystic portion of tumor ≥ 80%, incomplete tumor resection
Lee et al. (2022) [62]	Acute post-resection obstructive hydrocephalus and persistent hydrocephalus following acute hydrocephalus	NR	CPA tumors: vestibular schwannoma 64.9%, CPA meningioma 22.1%, petroclival meningioma 7.2%, trigeminal schwannoma 5.8%	154	Median 53 y (R 15–83)	Preop 19/154 (12.3%) Postop 17/154 (11%), 8/17 (47.1%) had transient hydrocephalus, 9/17 (52.9%) had persistent hydrocephalus and required VPS	Acute post-resection obstructive hydrocephalus: CPA meningioma, grade of tumor extension beyond the petroclival junction (grade 1 and 2), major fourth ventricle compression (grade 2), surgery-related hemorrhage Persistent hydrocephalus following acute hydrocephalus: preop hydrocephalus, surgery-related hemorrhage
Zhang C et al. (2022) [64]	Post-resection hydrocephalus and VPS	Excluded	Schwannoma 39.7%, meningioma 19.6%, hemangioblastoma 13.8%, glioma 8.7%, metastasis 4.3%, ependymoma 2.5%, medulloblastoma 1.2%, other 10.2%	675	Mean 48.6 y (R 19–83)	Preop 142/675 (21%) Postop acute hydrocephalus 47/675 (7%), 7/47 (14.9%) had persistent hydrocephalus and required VPS No postop acute hydrocephalus 638/675 (93%), subsequently 8/638 (1.3%) had persistent hydrocephalus and required VPS	Tumor infiltrating the ventricular system, postop hemorrhage
Darshan et al. (2023) [49]	Post-resection VPS	NR	Intraaxial tumor: metastasis 17.3%, hemangioblastoma 8.9%, pilocytic astrocytoma 6.7%, medulloblastoma 4.5%, other 7.7% Extraaxial tumor: schwannoma 33.9%, meningioma 18.8%, epidermoid cyst 2.2%	adult 313	Mean 48.7 ± 12.1 y	Preop 132/313 (42.2%) Postop VPS 82/313 (26.2%)	Intraaxial tumor: age. Frankfurt score, preop hydrocephalus, transependymal edema, EVD insertion, duration of EVD placement, tumor histology, extent of tumor resection Extraaxial tumor: sex, Frankfurt score, preop hydrocephalus, transependymal edema, EVD insertion, duration of EVD placement, EVD in situ, extent of tumor resection
		NR	Medulloblastoma 40.5%, pilocytic astrocytoma 35.3%, ependymoma 22.4%, glioblastoma 0.9%, choroid plexus papilloma 0.9%	Pediatric 116	Mean 9 ± 4.2 y	Preop 89/116 (76.7%) Postop VPS 38/116 (32.8%)	Sex, papilledema, EVD insertion, duration of EVD placement, EVD in situ
Hu et al. (2023) [63]	Post-resection hydrocephalus and VPS, and development of predictive model	Excluded	Medulloblastoma 39.6%, astrocytoma 34.1%, ependymoma 13.8%, other 12.5%	217	< 3 y: 34/217 (15.7%) 3–14 y: 183/217 (84.3%)	Preop 167/217 (76.9%), 68/167 (40.7%) underwent preop EVD Postop 29/217 (13.4%) required VPS	Age < 3 y (score of 2), intraop blood loss (score of 1), tumor at the fourth ventricle (score of 5) Patients with total scores ≥ 7.5 were classified as the high-risk group
Kumar et al. (2023) [96]	Post-resection permanent CSF diversion	Excluded	Medulloblastoma 51.9%, pilocytic astrocytoma 21.3%, ependymoma 19.4%, brainstem glioma (focal exophytic) 2.8%, choroid plexus papilloma 1.9%, other 2.8%	108	Median 9 y (R 1–16, IQR 7)	Preop 90/108 (83.3%) Postop permanent CSF diversion 42/108 (38.9%) (VPS 27/42, ETV 15/42)	Periventricular lucency (transependymal edema) on preop neuroimaging
Zhang N et al. (2023) [97]	Post-resection VPS	Excluded	Low-grade tumor 42.6%, high-grade tumor 57.4%	197	Median 5.4 y (R 0.3–14.2, IQR 2.7–8.2)	Preop moderate to severe hydrocephalus 52/197 (26.4%) Postop VPS 30/197 (15.2%)	Tumor metastasis, postop intraventricular blood
Zhang Z et al. (2023) [98]	Post-resection permanent CSF diversion	NR	Medulloblastoma 100%	131	Mean 6.5 ± 3.3 y	Preop EVD 110/131 (84%), no prevalence of hydrocephalus reported Postop permanent CSF diversion 34/131 (26%) (VPS 32/34, ETV 2/34)	Tumor volume > 46.4 cm^3^, CSF channel invasion
Zhou et al. (2023) [99]	Post-resection hydrocephalus and VPS	Excluded	Medulloblastoma: classic medulloblastoma 62.9%, desmoplastic/nodular medulloblastoma 24.8%, medulloblastoma with extensive nodularity 4.8%, large cell/anaplastic medulloblastoma 7.5%	105	Mean 7.6 y (R 1−18)	Preop 36/105 (34.3%) Postop 23/105 (21.9%), VPS 23/23 (100%)	Superior invasion, caudal invasion, intraventricular blood ≥ 5 mm
Amin et al. (2024) [100]	Post-resection hydrocephalus	Preop VPS 55/55 (100%)	Midline posterior cranial fossa tumor: astrocytoma 36.4%, medulloblastoma 29.1%, ependymoma 23.7%, choroid plexus papilloma 3.6%, epidermoid cyst 3.6%, meningioma 3.6%	55	Mean 9.5 ± 3.2 y (R 3–15)	Preop 55/55 (100%) Postop 3/55 (5.5%)	No variables were associated with post-resection hydrocephalus
Bernstein et al. (2024) [101]	Post-resection VPS	None	Metastasis 34.8%, meningioma 14.6%, hemangioblastoma 12.4%, astrocytoma 6.7%, schwannoma 5.6%, other 25.9%	89	Mean 52.8 ± 15.7 y	Preop NR Postop VPS 30/89 (33.7%), no shunt 40/89 (44.9%), unavailable data 19/89 (21.4%)	Postop CSF appearance being not clear, low CSF glucose level immediately before shunt placement
Samadder et al. (2024) [102]	Post-resection hydrocephalus	Preop VPS 43/43 (100%)	Midline posterior cranial fossa tumor 100% Tumor pathology: NR	43	Mean 8.3 ± 4.1 y	Preop 43/43 (100%) Postop 2/43 (4.7%)	No variables were associated with post-resection hydrocephalus
Obeng-Gyasi et al. (2025) [103]	Post-resection permanent CSF diversion	Excluded	Astrocytoma 39.9%, medulloblastoma 26.5%, ependymoma 15.9%, atypical teratoid/rhabdoid tumor 5.3%, hemangioblastoma 3.5%, brainstem glioma 1.8%, teratoma 0.9%, other 6.2%	113	< 2 y: 14/113 (12.4%) ≥ 2 y: 99/113 (87.6%)	Preop moderate to severe hydrocephalus 88/113 (77.9%) Postop permanent CSF diversion 35/113 (31%)	Moderate to severe hydrocephalus
Shabo et al. (2025) [65]	Post-resection hydrocephalus	None	Posterior cranial fossa metastasis 100%	130	Median 64 y (IQR 57–71)	Preop 63/130 (48.5%) Postop 14/130 (10.8%), transient hydrocephalus 8/14 (57.1%), persistent hydrocephalus and VPS 6/14 (42.9%)	A fourth-ventricle-to-tumor-volume ratio ≤ 0.02, an edema-to-tumor-volume ratio ≤ 0.85, tumors contact to the 4th ventricle on imaging, multiple intracranial metastases
The present study (2025)	Pre-resection hydrocephalus and development of predictive model	NR	Schwannoma 45.3%, malignant tumor 21%, meningioma 20.5%, other benign tumor 13.2%	421	Median 53 y (R 19–81)	Preop 160/421 (38%)	IICP symptom (score of 2), ataxia (score of 1), cognitive impairment (score of 4), tumor volume (score of 1 x tumor volume in cm^3^/10), peritumoral vasogenic edema (score of 3) Patients with total scores ≥ 4 were classified as the high-risk group with development of hydrocephalus

*AVM* arteriovenous malformation, *CPA* cerebellopontine angle, *CSF* cerebrospinal fluid, *CT* computerized tomography, *d* day, *ETV* endoscopic third ventriculostomy, *EVD* external ventricular drainage, *FOHR* frontal and occipital horn ratio, *h* hour, *ICP* intracranial pressure, *IICP* increased intracranial pressure, *intraop* intraoperative, *IQR* interquartile range, *IVH* intraventricular hemorrhage, *m* month, *NR* not reported, *periop* perioperative, *PNET* primitive neuroectodermal tumor, *postop* postoperative, *preop* preoperative, *R* range, *VAS* ventriculoatrial shunting, *VPS* ventriculoperitoneal shunting, *y* year

External ventricular drainage (EVD) is a temporary procedure of draining cerebrospinal fluid from the cerebral ventricles for relieving an elevated intracranial pressure

Frontal and occipital horn ratio (FOHR) is a measurement of ventricular size in pediatric patients with hydrocephalus. It was calculated by summation between maximum frontal horn diameter and maximum occipital horn diameter, then divided by twice the biparietal diameter. A normal FOHR is typically around 0.37

**Table 6:** Risk factors and predictors of hydrocephalus or the need for cerebrospinal fluid diversion in posterior cranial fossa tumors [6, 14, 21, 45–49, 59–65, 67, 69, 71, 73, 75–77, 80, 81, 83–85, 87, 89–99, 101, 103]

Risk factors/predictors of hydrocephalus or requirement of CSF diversion		Reference number				
		Pre-resection		Post-resection		
		Adult	Pediatric	Adult	Pediatric	
Clinical variables						
Young age					[14, 21, 46, 47, 63], [71]^c^, [73, 75, 81, 83, 85, 89–92]	
Old age		[60]^a^				
IICP symptom		Present study				
Ataxia		Present study				
Cognitive impairment		Present study				
Papilledema					[49, 83]	
Radiographic variables						
Large tumor size or volume		[59]^a^, [60]^a^, [61]^b^, present study		[48]	[98]^c^	
Midline tumor location				[67]	[21, 76, 81, 84]	
Tumor at the fourth ventricle					[63]	
Tumor contact to the fourth ventricle				[65]^e^		
Tumor extension				[62], [93]^f^	[71]^c^, [93]^f^, [99]	
Tumor compressing or infiltrating the fourth ventricle		[61]^b^		[62, 64]		
Cystic portion of tumor ≥ 80%				[69]^d^		
Peritumoral vasogenic edema		Present study		[67]		
Preop hydrocephalus				[47, 49, 62, 67], [69]^d^	[14], [71]^c^, [77, 81, 83, 84, 103]	
Transependymal edema				[48, 49, 67]	[14, 96]	
Brainstem compression				[47]		
A fourth-ventricle-to-tumor-volume ratio ≤ 0.02				[65]^e^		
An edema-to- tumor-volume ratio ≤ 0.85				[65]^e^		
Presence of metastasis or CSF dissemination					[83, 91, 97], [98]^c^	
Multiple brain metastases				[65]^e^		
Preop predicted tumor diagnosis (ependymoma, medulloblastoma or dorsally exophytic brainstem glioma)					[14, 83]	
Laboratory variables						
High CSF protein concentration		[80]^a^				
Low CSF glucose level before shunt placement				[101]		
Treatment variables						
Incomplete tumor resection				[49, 67], [69]^d^, [93]^f^	[21, 45, 46, 73, 85, 91, 92], [93]^f^	
Periop EVD insertion or CSF drainage				[48, 49]	[49, 75, 76, 84, 90, 92, 95]	
Prolonged EVD placement				[49]	[21, 49, 73, 92]	
Postop variables						
Early postop hydrocephalus					[90]	
Postop intraventricular blood				[48]	[89, 90, 97, 99]	
Postop hemorrhage				[62, 64]		
Postop infection or meningitis					[21, 84]	
Postop pseudomeningocele					[21, 84]	
Surgical complications				[48]		
Tumor pathology						
Medulloblastoma					[46, 47, 77, 84, 91]	
Ependymoma					[46, 75, 81, 84, 87, 94]	
Pilocytic astrocytoma				[47]		
Predictive score						
High mCPPRH score					[94]	
High Frankfurt score				[49]		

*CSF* cerebrospinal fluid, *EVD* external ventricular drainage, *IICP* increased intracranial pressure, *mCPPRH* Modified Canadian Preoperative Prediction Rule for Hydrocephalus, *periop* perioperative, *postop* postoperative, *preop* preoperative

^a^ Specific for pre-resection vestibular schwannoma in adult patients (clinical, radiographic, and laboratory variables)

^b^ Specific for pre-radio surgical vestibular schwannoma in adult patients (radiographic variable)

^c^ Specific for post-resection medulloblastoma in pediatric patients (clinical and radiographic variables)

^d^ Specific for post-resection vestibular schwannoma in adult patients (radiographic and treatment variables)

^e^ Specific for post-resection metastasis in adult patients (radiographic variable)

^f^ Specific for post-resection heterogeneous posterior cranial fossa tumor in mixed adult and pediatric age groups (radiographic and treatment variables)

Risk factors/predictors of hydrocephalus or requirement of CSF diversion in pre-resection and post-resection phases included clinical, radiographic, laboratory, treatment, and postoperative variables, tumor pathology, and predictive score

Several studies, including ours, identified large tumor size as a principal factor for pre-resection hydrocephalus in adults [59–61]. In contrast, tumor size was only linked to postoperative CSF diversion in one pediatric medulloblastoma study [98]. In children, post-resection hydrocephalus or the need for CSF diversion was commonly associated with young age [14, 21, 46, 47, 63, 71, 73, 75, 81, 83, 85, 89–92], midline tumor location [21, 75, 81, 84], preoperative hydrocephalus [14, 71, 77, 81, 83, 84, 103], metastatic or disseminated disease [83, 91, 97, 98], incomplete tumor resection [21, 45, 46, 73, 85, 91–93], perioperative external ventricular drainage (EVD) [49, 75, 76, 84, 90, 92, 95], prolonged EVD use [21, 49, 73, 92], intraventricular hemorrhage [89, 90, 97, 99], and specific pathologies such as medulloblastoma [46, 47, 77, 84, 91] or ependymoma [46, 75, 81, 84, 87, 94]. In adults, the principal predictors of post-resection hydrocephalus were preoperative hydrocephalus [47, 49, 62, 67, 69] and incomplete tumor resection [49, 67, 69, 93].

The present study has several strengths. First, we included consecutive cases across the study period, excluding those with multiple brain lesions, combined infratentorial and supratentorial tumors, or preexisting hydrocephalus before tumor diagnosis. This approach reduced selection bias and removed potential confounders. Second, our relatively large sample size mitigated concerns about inadequate numbers of participants, which have limited some earlier studies. Third, we developed a practical scoring model, derived from these data, to predict the likelihood of pre-resection hydrocephalus. Fourth, an extensive literature review and concise summary of risk factors or predictors of hydrocephalus or CSF diversion were also provided.

Despite these strengths, our study design remains susceptible to bias and confounding variables. In addition, we did not validate our scoring model in a separate patient cohort, and future work should address its external validity. Nonetheless, our findings may help neurosurgeons recognize clinical and radiographic signs that precede tumor-related hydrocephalus. The proposed predictive score can be used to identify patients at high risk for hydrocephalus, thus enabling earlier intervention. It may also guide surgical prioritization to prevent the devastating outcomes of severe hydrocephalus, including permanent neurologic deficits and death [104, 105].

## Conclusion

Clinical symptoms such as IICP symptom, ataxia, and cognitive impairment, along with radiographic findings (large tumor volume and peritumoral vasogenic edema), emerged as key predictors of pre-resection hydrocephalus in patients with posterior cranial fossa tumors. These insights can raise neurosurgeons’ awareness of hydrocephalus risk and guide the identification of high-risk patients for prompt surgical intervention to avert serious neurologic sequelae.

## Supplementary Information

Below is the link to the electronic supplementary material.

Supplementary Material 1 (DOCX 27.1 KB)

## Data Availability

No datasets were generated or analysed during the current study.
