# Supplementary material for: Predictors of pre-resection hydrocephalus in posterior cranial fossa tumors: development of a predictive scoring model
Source: Neurosurg Rev. 2025 Aug 19;48(1):607. doi: 10.1007/s10143-025-03752-2 (PMC12361321; doi:10.1007/s10143-025-03752-2)
Supplement: Supplementary file 1 — Supplementary Material 1 (DOCX 27.1 KB) [file 10143_2025_3752_MOESM1_ESM.docx]

**Supplementary Table** Receiver operating characteristic curve analysis for the predictive model

| **Cutoff total predictive score** | **Sensitivity (%)** | **Specificity (%)** | **PPV** | **NPV** | **LR+** | **LR-** | **AuROC** |
| --- | --- | --- | --- | --- | --- | --- | --- |
| ≥ 2.5 | 91.9 | 64.8 | 61.5 | 92.9 | 2.61 | 0.13 | 0.78 |
| ≥ 3 | 89.4 | 69.7 | 64.4 | 91.5 | 2.95 | 0.15 | 0.80 |
| ≥ 3.5 | 89.4 | 77.8 | 71.1 | 92.3 | 4.02 | 0.14 | 0.84 |
| ≥ 4 | 87.5 | 81.2 | 74.1 | 91.4 | 4.66 | 0.15 | 0.84 |
| ≥ 4.5 | 85.6 | 83.9 | 76.5 | 90.5 | 5.32 | 0.17 | 0.85 |

**Abbreviations:** AuROC, area under receiver operating characteristic curve; LR-, negative likelihood ratio; LR+, positive likelihood ratio; NPV, negative predictive value; PPV, positive predictive value
